# Establishment of inclusive single-cell transcriptome atlases from mouse and human tooth as powerful resource for dental research

**DOI:** 10.3389/fcell.2022.1021459

**Published:** 2022-10-10

**Authors:** Florian Hermans, Celine Bueds, Lara Hemeryck, Ivo Lambrichts, Annelies Bronckaers, Hugo Vankelecom

**Affiliations:** ^1^ Laboratory of Tissue Plasticity in Health and Disease, Cluster of Stem Cell and Developmental Biology, Department of Development and Regeneration, Leuven Stem Cell Institute, KU Leuven (University of Leuven), Leuven, Belgium; ^2^ UHasselt-Hasselt University, Biomedical Research Institute (BIOMED), Department of Cardio and Organ Systems, Diepenbeek, Belgium

**Keywords:** single-cell transcriptomics, tooth atlas, tooth development, tooth biology, stem cells

## Abstract

Single-cell (sc) omics has become a powerful tool to unravel a tissue’s cell landscape across health and disease. In recent years, sc transcriptomic interrogation has been applied to a variety of tooth tissues of both human and mouse, which has considerably advanced our fundamental understanding of tooth biology. Now, an overarching and integrated bird’s-view of the human and mouse tooth sc transcriptomic landscape would be a powerful multi-faceted tool for dental research, enabling further decipherment of tooth biology and development through constantly progressing state-of-the-art bioinformatic methods as well as the exploration of novel hypothesis-driven research. To this aim, we re-assessed and integrated recently published scRNA-sequencing datasets of different dental tissue types (healthy and diseased) from human and mouse to establish inclusive tooth sc atlases, and applied the consolidated data map to explore its power. For mouse tooth, we identified novel candidate transcriptional regulators of the ameloblast lineage. Regarding human tooth, we provide support for a developmental connection, not advanced before, between specific epithelial compartments. Taken together, we established inclusive mouse and human tooth sc atlases as powerful tools to potentiate innovative research into tooth biology, development and disease. The maps are provided online in an accessible format for interactive exploration.

## 1 Introduction

Teeth play crucial roles in life, being essential for eating, speaking and psychosocial wellbeing (Militi et al., 2021). Tooth pathologies, originating from a variety of causes such as traumatic injury, poor oral hygiene, and congenital disease, are highly prevalent with a large socio-economic burden (Dye, 2017; Kassebaum et al., 2017; Peres et al., 2019). Therefore, it is essential that tooth biology and pathology becomes more deeply understood, not only for fundamental insights but also for translational prospects toward better treatments through, among others, biological or bioengineered tooth replacements (reviewed in Angelova Volponi et al., 2018; Latimer et al., 2021; Pagella et al., 2021a).

In recent years, sc transcriptomic interrogations have been applied to mouse and human tooth. Regarding mouse, single-cell RNA-sequencing (scRNA-seq) datasets were generated from the constantly (re-)growing incisor and the more static, human-resembling molar at different timepoints, either of whole tooth or specific tissue components (Sharir et al., 2019; Takahashi et al., 2019; Chen et al., 2020; Chiba et al., 2020, 2021; Krivanek et al., 2020; Wen et al., 2020; Nagata et al., 2021; Zhao et al., 2021). Human sc transcriptomic data were predominantly generated from dental pulp and periodontal tissues of molars in both healthy and diseased states (Krivanek et al., 2020; Pagella et al., 2021b; Shi et al., 2021; Yin et al., 2021; Hemeryck et al., 2022; Lin et al., 2022; Opasawatchai et al., 2022). All these sc analyses generated deeper insight into the tooth molecular and cellular landscape, furthering the understanding of dental cell type heterogeneity and tooth biology. Among others, novel markers were identified for mouse dental epithelial and mesenchymal subsets allowing better discrimination of the cell populations (Sharir et al., 2019; Krivanek et al., 2020; Nagata et al., 2021) Similarly, in humans, the heterogeneity of dental pulp and periodontal mesenchymal (stem) cells was further elucidated (Krivanek et al., 2020; Pagella et al., 2021b; 2021c).

Within the research community, large efforts are being made in combining sc omics datasets to establish comprehensive cell atlases of all tissues and organs, both in healthy and diseased conditions (Regev et al., 2017; Han et al., 2018; Schaum et al., 2018; Almanzar et al., 2020), epitomized in large consortia such as the Mouse Cell Atlas (MCA) and Human Cell Atlas (HCA). In addition, the constant stream of progressing as well as new bioinformatic tools and pipelines allows deeper and innovative mining of these powerful datasets, thereby precipitating novel insights in tissue biology, development and disease.

At present, a comprehensive tooth atlas is not available yet. To address this important lacuna, we here set out to establish inclusive sc atlases of mouse and human tooth starting from recently published, publicly available sc transcriptome datasets, and to make these maps available online for easily accessible interactive interrogation. By applying state-of-the-art computational tools, we show that our newly consolidated tooth atlases are strongly applicable to retrieve novel insights in tooth biology and disease. Hence, the comprehensive and integrated atlases will provide a powerful tool to advance and enrich research into tooth development, biology and disease, and an essential cornerstone, both as driver and backing, for further hypothesis-driven research as well as for tooth bioengineering endeavors (Aibar et al., 2017; Efremova et al., 2020; Alquicira-Hernandez and Powell, 2021).

## 2 Materials and methods

### 2.1 Analysis of publicly available scRNA-seq datasets

Publicly available datasets used in this study were retrieved from the Gene Expression Omnibus (GEO), FaceBase, ArrayExpress and Mendeley Data databases (Tables 1, and 4). In general, deposited data, pre-processed by the originators, were available as count matrices. If not (as applying to Chen et al., 2020; Wen et al., 2020), raw sequencing data were processed using Cell Ranger (v3.1.0). All datasets were individually imported in Seurat (v4.0.0) for further downstream analyses (Stuart et al., 2019; Hao et al., 2021). Quality control was performed on each individual dataset. Low-quality cells and potential doublets were identified and removed based on number of counts and genes per cell as well as the percentage of mitochondrial genes expressed per cell (Tables 2 and 5).

**TABLE 1:** Publicly available mouse scRNA-seq datasets re-analyzed and integrated toward the comprehensive mouse tooth atlas.

Study	Accession number	Group	Tissue of interest	Age	Notes
Sharir et al. (2019)	GSE131204 (GEO)	Incisor	Dental epithelium	8–12 weeks	Only data generated from healthy controls were used
Takahashi et al. (2019)	GSE120108 (GEO)	Periodontal (molar)	Dental follicle	PD6	
Chen et al. (2020)	FB00001104 (FaceBase)	Incisor	Dental mesencyhme	4 weeks	
Chiba et al. (2020)	GSE146855 (GEO)	Incisor	Dental epithelium	PD7	
Krivanek et al. (2020)	GSE146123 (GEO)	Incisor, molar	Whole tooth	8–16 weeks	Only data produced with the 10X Genomics platform were included (GSM4365604, GSM4365605, GSM4365611)
Wen et al. (2020)	FB00001105 (FaceBase)	Molar	Whole tooth	PD7	Only data generated from healthy controls were used
Chiba et al. (2021)	GSE167989 (GEO)	Molar	Whole tooth	PD1	
Nagata et al. (2021)	GSE168450 (GEO)	Periodontal (molar)	Periodontal	PD25	
Zhao et al. (2021)	GSE160358 (GEO)	Periodontal (molar)	Periodontal	Adult	

Abbreviations: GEO, gene expression omnibus; wk, weeks; PD, postnatal day.

**TABLE 2:** Parameters used for quality control of the mouse tooth scRNA-seq datasets.

Study	nFeatures (range)	nCounts (cut-off)	% Mitochondrial genes (cut-off)	#Cells pre QC	# Cells post QC
Sharir et al. (2019)	[1,000:4,500]	<40,000	<5	3,599	3,093
Takahashi et al. (2019)	[1,000:6,000]	<40,000	<20	11,152	2,319
Chen et al. (2020)	[1,000:6,000]	<40,000	<20	7,413	3,634
Chiba et al. (2020)	[1,500:6,000]	<40,000	<5	6,260	6,062
Krivanek et al. (2020)	[750:2000]	<40,000	<15	4,236	869
Wen et al. (2020)	[1,000:6,000]	<40,000	<8	1844	1,205
Chiba et al. (2021)	[1,000:6,000]	<40,000	<20	4,293	2,553
Nagata et al. (2021)	[1,000:4,500]	<40,000	<5	6,010	5,842
Zhao et al. (2021)	[1,000:4,500]	<40,000	<5	6,064	5,872
Sharir et al. (2019)	[500:7,000]	<40,000	<20	8,073	4,854
Takahashi et al. (2019)	[1,000:7,000]	<40,000	<8	2,203	1,645

Abbreviations: QC, quality control.

### 2.2 Establishment of the mouse tooth atlas

Following quality control, mouse datasets were divided into three groups, i.e., incisor, molar and periodontal tissue, and datasets from each individual group were separately integrated. For each integrated group, after normalization and identification of variable features (using the NormalizeData and FindVariableFeatures functions), the FindIntegrationAnchors function was used with default parameters and dims = 1:30, and each group was integrated across all features using the IntegrateData function. Subsequently, expression levels were scaled and subjected to principal component analysis (PCA). Uniform Manifold Approximation and Projection (UMAP) dimensionality reduction was performed with the umap-learn package (v0.4.2) using the top 30 PC, after which clusters were determined with the FindClusters function using resolutions 0.3, 0.6 and 0.8 for the incisor, molar and periodontal groups, respectively (McInnes et al., 2018). Next, each group was roughly annotated before the count matrices were corrected for ambient/background RNA using the SoupX (v1.5.0) package (Young and Behjati, 2020). The global contamination fractions were estimated to be 4.7, 1 and 1% for incisor, molar and periodontal groups, respectively, well within the normal range (0–10%).

Then, all datasets were merged and, using the SoupX-corrected counts, integrated with the reciprocal PCA (rPCA) method which is a conservative tool that facilitates integration in case of many datasets and/or non-overlapping cell types (Hao et al., 2021). Therefore, after data normalization and identification of variable features, each individual dataset was scaled and subjected to PCA analysis, which was used as input to the FindIntegrationAnchors function (with dims = 1:30 and reduction = ‘rpca’) after which the datasets were integrated across all features using the IntegrateData function. During the scaling of the data, cell cycle regression was performed. Lists of human S and G2M genes were obtained from Seurat and converted to their mouse orthologues as input for the CellCycleScoring function. Following integration, the dataset was subjected to PCA, after which the top 50 PC were used for UMAP dimensionality reduction (with min. dist = 0.5) and clustering. The Seurat function AddModuleScore was used to evaluate the expression of gene marker profiles or ‘modules’. Each cell receives a module score based on the difference between the average expression of genes within the module and randomly selected control features.

### 2.3 Subclustering and pseudotime analysis of the mouse ameloblast lineage

The mouse ameloblast trajectory (encompassing the IEE-OEE, cycling DEP, DEP, preAB, sAB and mAB clusters) was extracted from the total mouse tooth atlas. Next, data integration was performed using the rPCA method on the SoupX-corrected counts as described above. Following integration, the top 40 PC were used for UMAP dimensionality reduction. Pseudotime analysis of the mouse ameloblast lineage was performed using Monocle3 (Trapnell et al., 2014; Qiu et al., 2017; Cao et al., 2019). The Monocle3 cell_data_set object was derived from the subclustered mouse ameloblast lineage Seurat object, including the computed UMAP dimensional reduction. Cells were ordered in pseudotime by manually selecting a root group within the IEE-OEE cluster. DEG along the pseudotime-ordered trajectory were computed using the Monocle3 graph_test function. Only DEG with Moran’s I > 0.1 and q < 0.01 were retained for further analysis. To create a heatmap of DEG along pseudotime using the ComplexHeatmap package (v2.10.0), genes were normalized using z-score transformation, and clustered using k-means clustering with k = 6 (Gu et al., 2016). GO analysis of DEG along the ameloblast trajectory was performed using Metascape (www.metascape.org) (Zhou et al., 2019).

### 2.4 Establishment of the human tooth atlas

Following quality control, the healthy or diseased human tooth datasets were provisionally integrated using the rPCA method, and cell cycle regression was performed (both as described above). Following integration, each integrated dataset was subjected to PCA (npcs = 100), after which the top 80 PC were used for UMAP dimensionality reduction and clustering. Next, the healthy or diseased human tooth datasets were corrected for ambient/background RNA using the SoupX (v1.5.0) package (1 and 9.8% estimated global contamination fraction, respectively). Then, the datasets were definitively integrated using the rPCA method, and cell cycle regression was performed. Following integration, the dataset was subjected to PCA (npcs = 100), after which the top 80 PC were used for UMAP dimensionality reduction. Using the FindClusters function and evaluation of marker profiles, the distinct clusters were annotated.

### 2.5 Integration of healthy and diseased human tooth atlases

Finalized healthy and diseased human tooth atlases were merged, and integrated using the rPCA method (using npcs = 30 and approx = FALSE). Following integration, the dataset was subjected to PCA (npcs = 100), after which the top 80 PC were used for UMAP dimensionality reduction (with min. dist = 0.5).

### 2.6 Subclustering of human dental epithelium

Human ‘Epithelial’ and ‘Cycling’ clusters were extracted from the main Seurat object and subjected to rPCA-based integration (using the SoupX-corrected count matrices; with non-default RunPCA parameters npcs = 30 and approx = FALSE), PCA analysis, UMAP dimensionality reduction (using the top 30 PC), clustering (resolution 1.2) and annotation based on marker expression profiles and tissue of origin.

### 2.7 Integration of mouse and human dental epithelium

All mouse DE clusters were extracted from the main mouse tooth atlas. Annotated human DE clusters, excluding the ‘Cycling’ group, were extracted from the DE-subclustered healthy human tooth atlas. Human gene names were converted to their mouse orthologues. Integration was performed using the rPCA method as described above, using default parameters. Subsequently, the integrated dataset was subjected to PCA (npcs = 30), after which the top 40 PC were used for UMAP dimensionality reduction.

### 2.8 Weighted kernel density estimation of gene expression using nebulosa

The Nebulosa (v1.0.2) package was used to perform (joint) weighted kernel density estimation of gene expression using the plot_density function with default parameters (Alquicira-Hernandez and Powell, 2021).

### 2.9 Differentially expressed gene analysis

DEG analysis was performed in Seurat with the SoupX-corrected counts using the FindAllMarkers function with logfc. threshold = 0.25 and min. pct = 0.25.

### 2.10 Analysis of gene-regulatory networks

GRN (or regulons) were identified using pySCENIC (v.0.9.15) as described before (Vennekens et al., 2021). In short, co-expression modules were generated, and regulons inferred with default parameters. For mouse, mm10__refseqr80__10 kb_up_and_down_tss.mc9nr and mm10__refseqr80__500 bp_up_and_100 bp_down_tss.mc9nrmotif collections were used. The hg38__refseq-r80__10 kb_up_and_down_tss.mc9nr.feather and hg38__refseq-r80__500 bp_up_and_100 bp_down_tss.mc9nr.feather motif collections were used for human data. The analysis results in a matrix of AUCell values that represent the activity of each regulon in each cell. Using the AUCell matrix as input, the datasets were re-integrated using Seurat and subjected to PCA analysis and UMAP dimensional reduction. For the complete mouse atlas, the top 15 PC were used. The AUCell matrix was imported into the original integrated Seurat objects, and regulons were projected on the integrated UMAP plots. Regulon specificity scores (RSS), i.e., the cell-type specificity of a regulon, were calculated using the SCENIC function regulon_specificity_scores (Suo et al., 2018). Mean regulon activity (MRA) was calculated as the mean activity of each regulon per cluster. Min-max normalization was used to scale each parameter. Both scores were co-assessed by scatter plots of both metrics, and by multiplying RSS with the MRA for each regulon, allowing the ranking of regulons based on both parameters.

### 2.11 Inference of ligand-receptor interactions using CellPhoneDB

The CellPhoneDB package (v2.1.5) was used to project ligand-receptor interactions between annotated cell types using the SoupX-filtered and normalized count matrices as input and with default parameters (Efremova et al., 2020). Mouse gene names were converted to their human orthologues prior to running CellPhoneDB analysis.

### 2.12 Establishment of loom files for interactive exploration in SCope

Loom files were established from the mouse atlas (as well as from its subclustered DE) and from the human healthy and diseased atlases (as well as from the integrated healthy and diseased atlas, and from the subclustered DE) using Loompy v2.0.17 (Linnarsson lab, www.loompy.org). Loom files can be uploaded into SCope (available from https://scope.aertslab.org), where they can be interactively explored (Davie et al., 2018).

## 3 Results and discussion

### 3.1 Establishing a comprehensive single-cell atlas of mouse tooth

**FIGURE 1:**
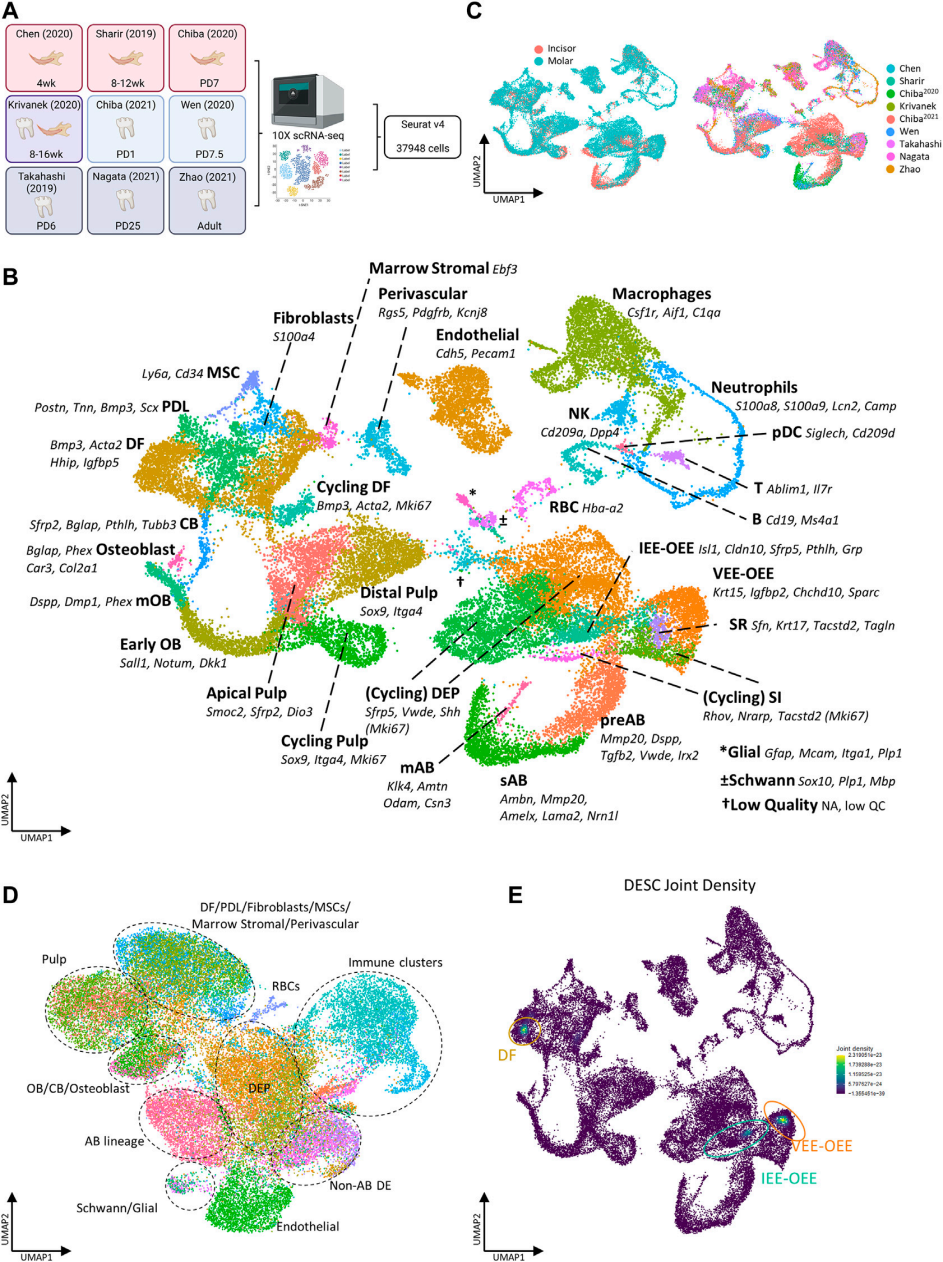
Establishment of an inclusive mouse tooth single-cell atlas. **(A)** Schematic overview of workflow and datasets incorporated in the mouse tooth atlas with tooth type and animal age. Colors indicate the tissue types derived from each dataset (red, incisor; blue, molar; purple, incisor and molar; gray, periodontal). Abbreviations: wk, weeks; PD, postnatal day. **(B,C)** UMAP plots of annotated clusters with top marker genes for each cluster **(B)**, and of tooth type and dataset **(C)**. **(D)** UMAP plot displaying regulon-based clustering of the mouse tooth atlas. Dotted lines indicate the overarching cell groups identified. **(E)** UMAP plots showing the joint Nebulosa expression densities for the DESC marker panel (consisting of *Sox2*, *Lgr5*, *Gli1*, *Lrig1*, *Bmi1*, *Ptch1*, *Sfrp5*, *Pcp4*, *Pknox2*, *Zfp273* and *Spock1*, see text and Supplementary Figure S1G).

With the aim of establishing an inclusive sc atlas of postnatal mouse teeth, we set out to integrate the at present nine publicly available scRNA-seq datasets (Figure 1A; Table 1) (Sharir et al., 2019; Takahashi et al., 2019; Chen et al., 2020; Chiba et al., 2020; Krivanek et al., 2020; Wen et al., 2020; Chiba et al., 2021; Nagata et al., 2021; Zhao et al., 2021). After rigorous quality control (i.e., removal of low-quality cells and doublets), 37,948 cells were retained for further analysis, with the datasets contributing between 1,645 and 11,714 cells to the total pool, and with 24,290 and 13,658 cells obtained from molars and incisors, respectively (Supplementary Figures S1A,B, Table 2). Based on expression of known markers and marker profiles (‘modules’) established from literature, as well as of novel cell type markers identified in the original scRNA-seq datasets, 35 distinct cell clusters were annotated (Figure 1B, Supplementary Figures S1C,D, Table 3). Cells from each dataset (i.e., from the different tooth (tissue) types and across timepoints) nicely integrated in their respective clusters (Figure 1C).

**TABLE 3:** Gene panels used for cluster annotation of mouse tooth cell types.

Cluster	Genes	Cluster	Genes
mAB	*Klk4, Amtn, Odam, Csn3*	DF	*Bmp3, Acta2, Igfbp5, Tnmd, Spon1, Hhip*
sAB	*Ambn, Amelx, Mmp20, Enam, Lama2, Nrn1l, Cd55, Plod2, Galnt12, Cd24a*	Cycling DF	*Bmp3, Acta2, Igfbp5, Tnmd, Spon1, Hhip, Mki67, Top2a*
preAB	*Amelx, Mmp20, Dspp, Tgfb2, Vwde, Irx2, Mme, Cyp26a1, Shh, Igfbpl1, Col22a1*	Fibroblasts	*S100a4, Spon2*
DEP	*Sfrp5, Vwde, Cyp26a1, Shh, Igfbpl1, Col22a1, Kif5c*	Marrow stromal	*Ebf3*
Cycling DEP	*Sfrp5, Vwde, Cyp26a1, Shh, Igfbpl1, Col22a1, Kif5c, Mki67, Top2a*	MSC	*Foxd1, Cd34, Ly6a*
VEE-OEE	*Krt15, Igfbp2, Chchd10, Sparcl1, Cltb, Notch2, Igfbp7, Gas6, Sparc*	Perivascular	*Rgs5, Pdgfrb, Kcnj8*
IEE-OEE	*Isl1, Cldn10, Sfrp5, Pthlh, Grp, Shisa2, Crabp1, Fam19a4, Shh, Aplf, Krt18,*	Endothelial	*Pecam1, Emcn, Col4a1, Cdh5*
SR	*Krt17, Tacstd2, Sfn, Tagln, Igfbp3, Pthlh*	B	*Ptprc, Cd19, Ms4a1*
SI	*Rhov, Nrarp, Tacstd2, Krt17, Cldn10, Enpp2*	T	*Ptprc, Il7r, Ablim1, Cd8a*
Cycling SI	*Rhov, Nrarp, Tacstd2, Krt17, Cldn10, Mki67, Enpp2, Top2a*	pDC	*Siglech, Cd209d*
mOB	*Dspp, Dmp1, Phex, Cox4i2, Nupr1, Bglap*	Neutrophil	*S100a8, S100a9, Camp, Lcn2*
Early OB	*Sall1, Notum, Dkk1, Smpd3, Bglap*	NK	*Cd209a, Cd209c, Dpp4*
Apical pulp	*Sfrp2, Dio3, Smoc2, Shisa2, Hhip*	Macrophage	*Csf1r, Aif1, C1qa, Lyve1*
Distal pulp	*Sox9, Itga4, Bglap*	Schwann	*Plp1, Cnp, Mag, Sox10, Mbp, S100b*
Cycling pulp	*Sox9, Itga4, Sostdc1, Mki67, Top2a*	Glial	*Plp1, Sox10, Itga1, Mcam, Gfap, S100b*
CB	*Pthlh, Smpd3, Sparcl1, Spp1, Olfml2b, Tubb3, Omd, Ibsp, Dmp1, Bglap, Postn, S100a4, Sfrp2, Col12a1*	RBC	*Hba-a2*
Osteoblast	*Col2a1, Car3, Phex, Bglap, Col22a1*	Low Quality	No clear markers, low QC metrics
PDL	*Scx, Tnn, Postn, Bmp3*		

Regarding the epithelial compartment, which comprises 36.9% of all annotated cells, 10 distinct dental epithelium (DE) lineages were identified: DE progenitors (DEP), cycling DEP, pre-ameloblasts (preAB), secretory-stage ameloblasts (sAB), maturation-stage ameloblasts (mAB), stratum intermedium (SI), cycling SI, stellate reticulum (SR), ventral/outer enamel epithelium (VEE-OEE) and inner/outer enamel epithelium (IEE-OEE) (Figure 1B, Supplementary Figures S1E,F). Concerning the mesenchymal part, containing 38.4% of classified cells, 13 dental mesenchyme (DM) clusters were catalogued: apical pulp, distal pulp, cycling pulp, early odontoblasts (OB), mature OB (mOB), cementoblasts (CB), osteoblasts, dental follicle (DF), cycling DF, periodontal ligament (PDL), fibroblasts, mesenchymal stem cells (MSC) and marrow stromal cells (Figure 1B, Supplementary Figure S1E). Further designated clusters encompass endothelial, perivascular, red blood (RBC), Schwann and glial cells, and a large population (14.4%) of immune cells (Figure 1B, Supplementary Figure S1E). The latter is subdivided in macrophages, neutrophils, B, NK and T cells, and plasmacytoid dendritic cells (pDC). Finally, a small cluster containing remnant low-quality cells originating from all nine datasets was distinguished, which could not be clearly annotated. Of note, clustering based on gene regulatory networks (GRN; i.e., regulons, being modules of a core transcription factor (TF) with its predicted target genes co-expressed in the same cell) as identified using the pySCENIC pipeline (Aibar et al., 2017), revealed highly similar regulatory landscapes of cell clusters within their respective overarching cell group (e.g., co-clustering of apical with distal and cycling pulp ito ‘pulp’, or of preAB with sAB and mAB into ‘AB lineage’) (Figure 1D). Taken together, through aggregating recently published, publicly available sc transcriptomic datasets of mouse molars and incisors, a comprehensive sc transcriptome atlas of mouse tooth could be established, capturing its cellular diversity. This atlas provides an interesting resource to advance research into mouse tooth biology which can be exploited using state-of-the-art bioinformatic tools, as demonstrated below.

### 3.2 Identifying projected dental epithelial stem cells using the newly built mouse tooth atlas

Using the above applied standard bioinformatic approaches, we could not identify a distinct dental epithelial stem cell (DESC) cluster in the newly composed mouse tooth atlas (Figure 1B). This observation corresponds to previous reports which also could not pinpoint a DESC cluster in their datasets (Sharir et al., 2019; Chiba et al., 2020; reviewed in Fresia et al., 2021). One study, which also applied the Smart-seq2 platform yielding higher-depth sequencing data than 10X Genomics, was able to identify putative DESC clusters (Krivanek et al., 2020). DESC have been reported to represent a mixed population, characterized by (combinations of) expressed markers such as *Sox2*, *Lgr5*, *Gli1*, *Lrig1*, *Bmi1* and *Ptch1* (Seidel et al., 2010, 2017; Juuri et al., 2012; Biehs et al., 2013; Sanz-Navarro et al., 2018; Hermans et al., 2021). Since one of the limitations of sc transcriptomics, especially when sequenced at lower depth, is the occurrence of ‘dropouts’ (i.e. absence of parts of the cell’s transcriptome due to low mRNA expression and/or inefficient mRNA capture (Qiu, 2020)), we applied a novel bioinformatic tool, i.e., weighted kernel density estimation as implemented in the Nebulosa package, which endeavors to recover signals (‘densities’) from dropped-out features (in other words, ‘rescuing’ expression signals of dropped-out genes) through incorporating cellular similarities (Alquicira-Hernandez and Powell, 2021). The individually estimated gene densities can then be aggregated into a joint density for the panel of assessed genes. Determining the individual densities of well-reported DESC markers (*Sox2*, *Lgr5*, *Gli1*, *Lrig1*, *Bmi1*, *Ptch1*), together with those of the DEP indicator *Sfrp5* (Juuri et al., 2012) and of potential novel markers identified through deep-sequencing interrogation (*Pcp4*, *Pknox2*, *Zfp273*, *Spock1* (Krivanek et al., 2020)) (Supplementary Figure S1G), followed by their assembling into joint density, revealed putative DESC localized within the IEE-OEE and VEE-OEE clusters (Figure 1E), consistent with the most recent DESC projections (Gan et al., 2020; Fresia et al., 2021; Hermans et al., 2021). Because a number of the applied markers may also denote DM stem cells, the weighted kernel density estimation also identified a potential stem cell cluster within the DF (Biehs et al., 2013; Seidel et al., 2017; Hermans et al., 2021). Taken together, by applying the novel bioinformatic density estimation tool on our newly composed atlas, we were able to discern projected DESC clusters using datasets which previously did not reveal these cells, hence showing the high potential of the inclusive mouse tooth sc atlas by elevating the analytical power.

### 3.3 Leveraging the mouse tooth atlas to decipher the ameloblast lineage

**FIGURE 2:**
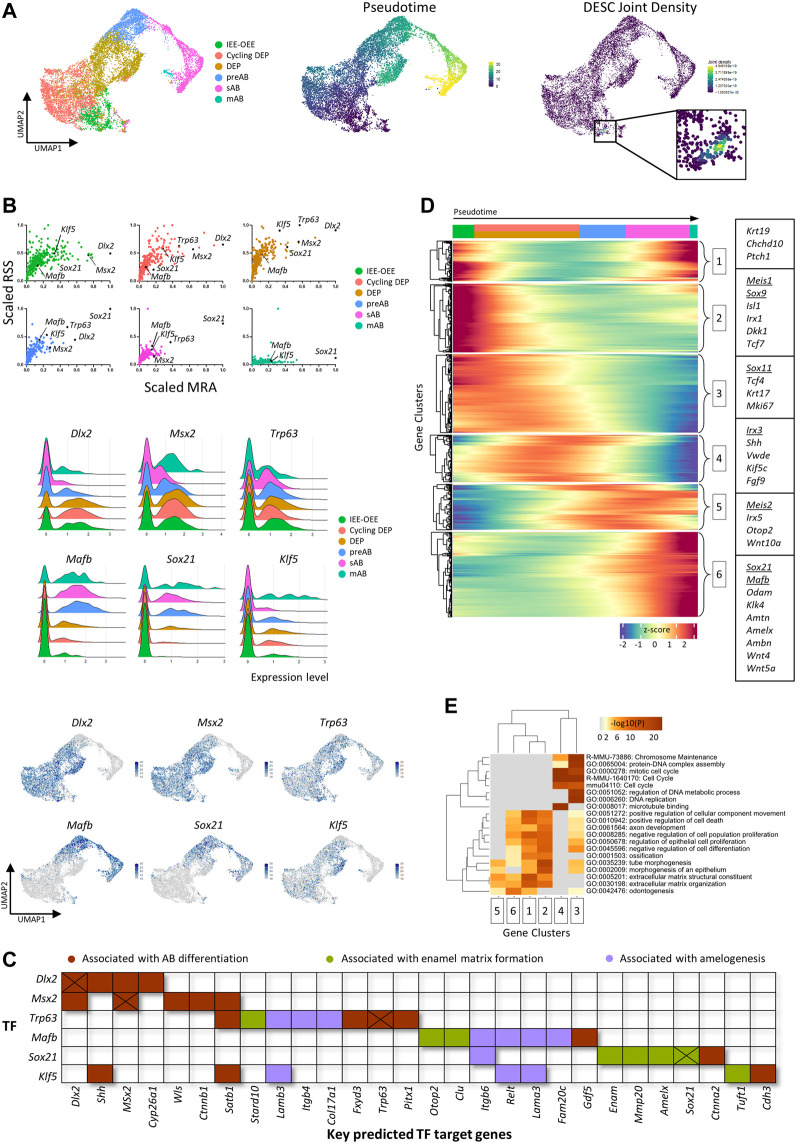
Subclustering of the mouse ameloblast lineage. **(A)** UMAP plots of subclustered ameloblast lineage (left), pseudotime trajectory (starting from the IEE-OEE cluster; middle) and joint Nebulosa expression density for the DESC marker panel (right). **(B)** Scatter plots (per annotated epithelial cell cluster, color-coded) of scaled RSS and MRA with each dot corresponding to an individual regulon and black dots indicating the marked regulons (top), and ridge (middle) and feature (bottom) plots indicating gene expression of selected TF/regulons. **(C)** Map indicating key predicted target genes of identified TF. Genes are color-coded based on association with AB differentiation (brown), enamel matrix formation (green) or amelogenesis (purple). Cross marks indicate potential self-Induction/upregulation. **(D)** Pseudotime-ordered (from left to right) gene expression heatmap of the ameloblast trajectory in panel **(A)**. Color intensity indicates z-score normalized gene expression (legend at bottom). Genes were clustered into six groups using k-means clustering. Boxes indicate examples of genes for each cluster (in-text mentioned genes are underlined). **(E)** Heatmap of GO analysis on clustered genes from panel **(D)**. Color intensity indicates the *p* values of displayed GO terms (legend on top).

We explored whether the created tooth sc atlas was instrumental to delve into the still poorly understood AB lineage and differentiation. Subclustering and pseudotime analysis using Seurat and Monocle projected a linear trajectory starting from the IEE-OEE cluster toward the cycling DEP, DEP, preAB, sAB and mAB clusters (Figure 2A). Again, weighted kernel density estimation of the DESC marker panel applied to this focused dataset designated the IEE-OEE cluster to contain the putative DESC (Figure 2A).

Our initial GRN analysis revealed a highly similar regulatory landscape among related DE cell types, as indicated by joint clustering of, amongst others, preAB, sAB and mAB (‘AB lineage’); DEP and cycling DEP (‘DEP’); and non-AB epithelium (‘Non-AB DE’) (see Figure 1D). By combining regulon specificity score (RSS), mean regulon activity (MRA) and expression pattern of regulon TFs, to our knowledge in such combination previously not performed, candidate transcriptional regulators across the AB differentiation trajectory could be identified, with dynamic gene expression patterns and varying degrees of regulon specificity and activity along the trajectory (Figure 2B). Whereas *Dlx2* and *Msx2* are predominantly expressed in the developmentally early IEE-OEE and (cycling) DEP, and *Trp63* is more constant throughout the trajectory (albeit with slowly declining expression and regulon activity), the applied metrics advanced *Mafb* and *Sox21* as strong-candidate transcriptional regulators of the AB lineage. *Mafb* and *Sox21* share a similar expression pattern, being upregulated from the (late) DEP stage onward (Figure 2B). Also, *Klf5* was found to display an intriguing expression profile and regulon specificity/activity, being prominent throughout AB development except during the secretory stage (Figure 2B). The projected target genes of the characterized TF, as determined by the pySCENIC pipeline (using co-expression and binding motif analysis), include key factors associated with AB differentiation, enamel matrix formation and amelogenesis (Figure 2C, Supplementary Figure S2A). For example, *Satb1*, a key regulator of ameloblast polarity, is projected to be regulated by *Msx2* and *Klf5*. Furthermore, target genes of *Mafb* include the amelogenesis-associated genes *Itgb6* and *Lama3*. Congenital mutations in laminins (*Lama3* as well as *Lamb3*, target gene of *Klf5*) are associated with *amelogenesis imperfecta*. Target genes of *Sox21* encompass the enamel matrix proteins *Enam* and *Amelx*, as well as *Mmp20* and *Sox21* itself (suggesting a positive feedback loop, as also seen for *Dlx2*, *Msx2* and *Trp63*). Interestingly, *Sox21* has recently been independently identified as a key regulator of AB identity and function (Saito et al., 2020).

Assessment of differentially expressed genes (DEG) across the pseudotime-ordered trajectory provides another tool to monitor and identify candidate regulators of the AB lineage (Figure 2D). For example, a shift in TF expression is observed from *Meis1*, *Sox9* and *Sox11* in DEP towards *Irx3*, *Meis2*, *Sox21* and *Mafb* in committed and differentiating AB (Figure 2D). Of note, gene ontology (GO) exploration of the DEG shows an evolution from cell cycle-associated processes (e.g., ‘DNA replication’, ‘DNA strand elongation’, ‘mitotic cell cycle’) to differentiation-linked terms (e.g., ‘odontogenesis’, ‘extracellular matrix organization’, ‘biomineral tissue development’), concordant with the projected AB development trajectory (Figure 2E, Supplementary Figure S2B).

Taken together, the inclusive mouse tooth cell atlas composed here provides a valuable tool to generate new insights, thereby opening the path toward deeper understanding of mouse tooth biology and development. For instance, the novel regulators of AB differentiation surfacing from the bioinformatic analysis of the atlas can now be further examined *in vivo* (e.g., using genetically modified mice) or *in vitro* (e.g., using CRISPR/Cas gene editing in tooth organoid models (Hemeryck et al., 2022)). Detailed mapping of interactions between cell types (e.g., DE and DM) using the new atlas is expected to shed light on tooth development which is known to encompass key epithelial-mesenchymal interactions underlying the joint differentiation processes of OB and AB (Hermans et al., 2021). Once gathered, this knowledge can be used to compose *in vitro* (complex organoid) models that capture the odontogenesis and amelogenesis processes. Such study models are currently lacking, thereby obstructing tooth mineralization research. Establishing such biomimetic systems would open up the avenue to a wide array of aspirations, from elucidating congenital tooth disorders to accomplishing bioengineered tooth replacement strategies. Of note, the currently existing mouse scRNA-seq studies provide fragmented information regarding tooth/tissue type and age/developmental stage, which may still hinder straightforward in-depth comparisons. Hence, a more systematic sampling of several different dental tissues across consecutive postnatal timepoints would be highly valuable (as recently reported for mouse embryonic molar and incisor tooth germs (Wang et al., 2022)).

### 3.4 Establishing a comprehensive atlas of adult human tooth

**FIGURE 3:**
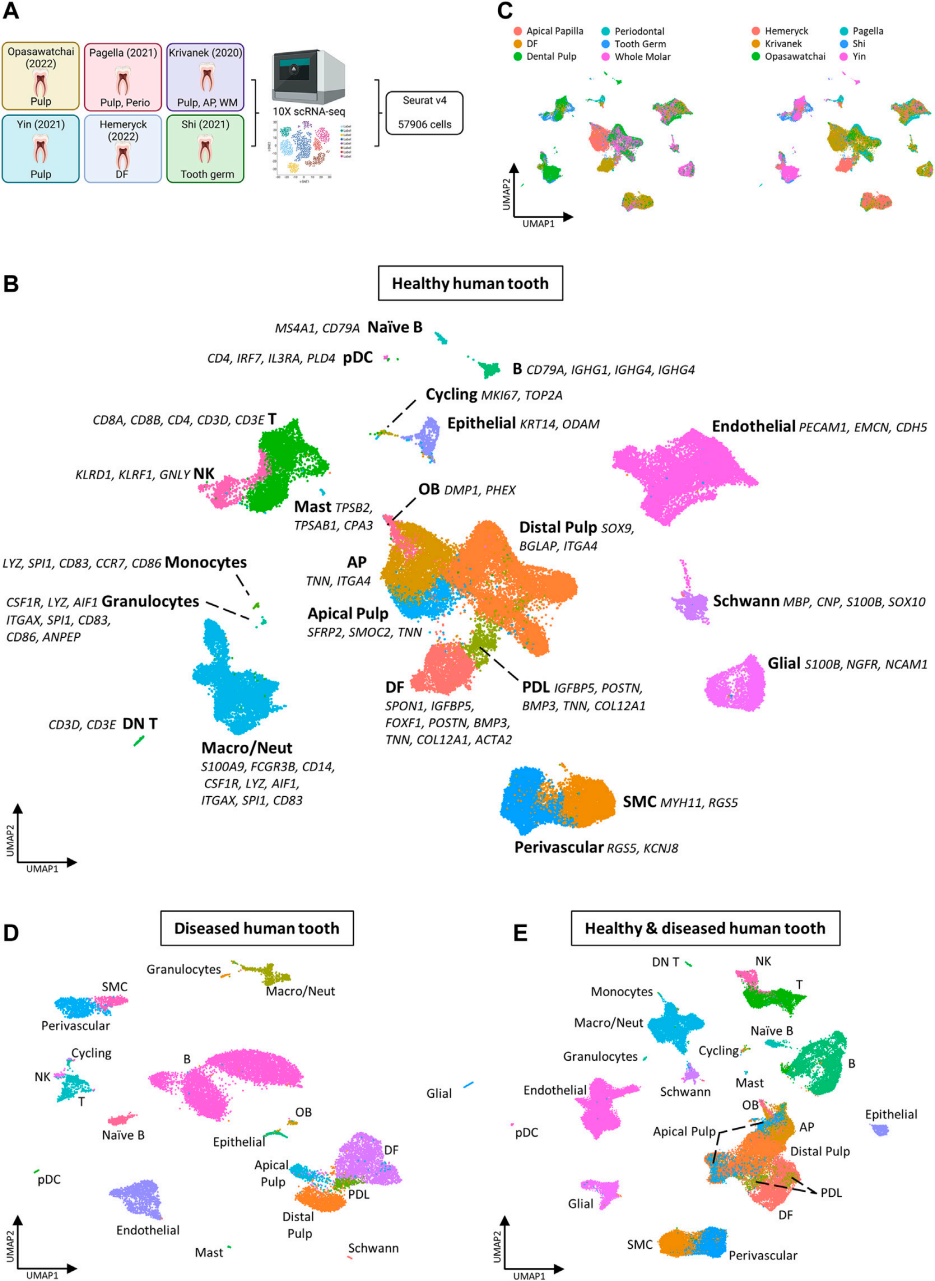
Establishment of an inclusive human tooth single-cell atlas. **(A)** Schematic overview of workflow and datasets incorporated in the healthy human tooth atlas with tooth (being molar) and tissue type. Abbreviations: perio, periodontal; AP, apical papilla; WM, whole molar; DF, dental follicle. **(B–E)** UMAP plots of the annotated clusters of the healthy human tooth atlas with top marker genes for each cluster **(B)**, of tissue type and dataset **(C)**, of the annotated diseased tooth atlas **(D)**, and of the integrated healthy and diseased tooth atlas **(E)**.

To complement the mouse tooth sc atlas, we also set out to establish an inclusive sc transcriptomic atlas of healthy human teeth (molars) by combining the at present six publicly available datasets (Figure 3A; Table 4) (Krivanek et al., 2020; Pagella et al., 2021b; Shi et al., 2021; Yin et al., 2021; Hemeryck et al., 2022; Opasawatchai et al., 2022). Following stringent quality control, 57,906 cells were found suitable for further bioinformatic analysis, with the datasets contributing between 524 and 21,453 cells to the total set, and cells sampled from various dental tissue types (e.g., 24,412 cells from DP and 7,119 from DF) (Supplementary Figures S3A,B, Table 5). Mapping established marker profiles resulted in the annotation of 23 distinct cell clusters (Figure 3B, Supplementary Figure S3C, Table 6). Cells from each dataset and the corresponding different tissue parts appropriately integrated (Figure 3C). Within the mesenchymal compartment, comprising 38% of total cells, six cell clusters were pinpointed, i.e., distal pulp, apical papilla (AP), DF, apical pulp, PDL and OB (Figure 3B, Supplementary Figure S3D). Of note, only one small epithelial cluster (1.1% of total cells) was discerned (Figure 3B, Supplementary Figure S3D), in clear contrast with the multitude of DE clusters surfacing in the mouse atlas, which may be due to the overrepresentation of mesenchymal input tissues (in particular, dental pulp) in the human datasets (Supplementary Figure S3B, Table 4), but also the different biology of human and mouse tooth, the latter being highly dynamic in turnover while human tooth loses the preponderance of DE cells upon completion of crown development. Similarly, only a limited number of OB were found (0.9% of total cells versus 5.3% mOB and early OB in the mouse atlas), likely due to completion of dentinogenesis in the collected human tooth samples and/or limited number of periodontal tissues in the composed human atlas’ dataset. In accordance, periodontal CB were not detected (Figure 3B; Supplementary Figure S3D). On the other hand, endothelial, smooth muscle (SMC), perivascular, glial, Schwann and cycling cells could be discriminated (Figure 3B; Supplementary Figure S3D).

**TABLE 4:** Publicly available human scRNA-seq datasets re-analyzed and integrated toward the comprehensive human tooth atlas.

Study	Accession number	Group	Tissue of interest	Age	Notes
**Healthy human**					
Krivanek et al. (2020)	GSE146123 (GEO)	Molar	Dental pulp, apical papilla, Whole molar	18–31 yo	
Pagella et al. (2021b)	GSE161267 (GEO)	Molar	Dental pulp, periodontium	18–35 yo	
Shi et al. (2021)	NA	Molar	Tooth germ	Unknown	Data are publicly available on Mendeley Data via https://data.mendeley.com/datasets/7ryrp25y6z
Yin et al. (2021)	GSE167251 (GEO)	Molar	Dental pulp	15 yo	
Hemeryck et al. (2022)	E-MTAB-10596 (ArrayExpress)	Molar	Dental follicle	15–18 yo	
Opasawatchai et al. (2022)	GSE185222 (GEO)	Molar	Dental pulp	21 yo	Only data generated from healthy controls were used
**Diseased Human**					
Lin et al. (2022)	GSE181688 (GEO)	Molar	Periapical tissue	26–44 yo	CAP, periapical granuloma
Opasawatchai et al. (2022)	GSE185222 (GEO)	Molar	Dental pulp	20–36 yo	Enamel caries, deep caries

Abbrevations: GEO, gene expression omnibus; yo, years old; CAP: chronical apical periodontitis.

**TABLE 5:** Parameters used for quality control of the healthy human tooth scRNA-seq datasets.

Study	nFeatures (range)	nCounts (cut-off)	% Mitochondrial genes (cut-off)	#Cells pre QC	# Cells post QC
**Healthy human**					
Krivanek et al., 2020 (dental pulp)	[400:1,500]	<40,000	<5	3,386	2,632
Krivanek et al., 2020 (apical papilla)	[500:1,500]	<40,000	<6	13,830	9,936
Krivanek et al., 2020 (whole tooth)	[1,000:3,000]	<40,000	<10	23,178	8,885
Pagella et al. (2021b) (pulp)	[800:3,000]	<40,000	<15	32,851	15,524
Pagella et al. (2021b) (periodontal)	[750:4,000]	<40,000	<10	2,914	1,590
Shi et al., 2021 (tooth germ)	[750:2,500]	<40,000	<20	9,855	5,964
Yin et al., 2021 (dental pulp)	[500:3,000]	<40,000	<15	7,121	5,732
Hemeryck et al., 2022 (dental follicle)	[450:5,500]	<40,000	<10	7,891	7,119
Opasawatchai et al., 2022 (dental pulp)	[900:3,000]	<40,000	<3	890	524
**Diseased Human**					
Lin et al., 2022 (CAP)	[1,000:3,500]	<40,000	<6	19,089	10,540
Lin et al., 2022 (periapical granuloma)	[1,100:3,500]	<40,000	<5	7,265	2,914
Opasawatchai et al., 2022 (deep caries)	[900: 3,500]	<40,000	<3	3,677	2,586
Opasawatchai et al., 2022 (enamel caries)	[800: 3,500]	<40,000	<3	2015	976

Abbreviations: CAP, chronical apical periodontitis; QC, quality control.

**TABLE 6:** Gene panels used for cluster annotation of human tooth cell types.

Cluster	Genes	Cluster	Genes
Epithelial	*KRT14, ODAM*	B	*CD79A, IGHG1, IGHG4, IGHG4*
Cycling	*KRT14, ODAM, TOP2A*	DN T	*CD3D, CD3E, IL4 (CD4* ^*−*^ *, CD8* ^*−*^ *)*
OB	*DMP1, PHEX*	T	*CD3D, CD3E, CD4, CD8A, CD8B*
Distal pulp	*SOX9, BGLAP, ITGA4*	NK	*KLRD1, KLRF1, GNLY*
Apical pulp	*SFRP2, SMOC2, TNN*	pDC	*CD4, IRF7, IL3RA, PLD4*
AP	*TNN, ITGA4*	Macro/Neut	*S100A9, FCGR3B, CD14, CSF1R, LYZ, AIF1, ITGAX, SPI1, CD83*
DF	*SPON1, IGFBP5, FOXF1, POSTN, BMP3, TNN, COL12A1, ACTA2*	Monocytes	*LYZ, SPI1, CD83, CCR7, CD86*
PDL	*IGFBP5, POSTN, BMP3, TNN, COL12A1*	Granulocytes	*CSF1R, LYZ, AIF1, ITGAX, SPI1, CD83, CD86, ANPEP*
SMC	*MYH11, RGS5*	Mast	*TPSB2, TPSAB1, CPA3*
Perivascular	*RGS5, KCNJ8*	Schwann	*MBP, CNP, S100B, PLP1, SOX10*
Endothelial	*PECAM1, EMCN, CDH5*	Glial	*S100B, PLP1, SOX10, FOXD3, NCAM1, NGFR*
Naïve B	*MS4A1, CD79A*		

Regarding the immune component, the composed human tooth atlas revealed a larger population than exposed in mouse tooth (23.5% versus 14.4%, respectively), moreover showing a distinct composition (Supplementary Figure S3E). Overall, the proportions of immune cells as surfacing here in the human atlas are comparable to previously reported flow-cytometric data (in particular derived from dental pulp) (Gaudin et al., 2015). Several immune cell types were identified in human but not mouse tooth including mast cells, monocytes, granulocytes, double negative (DN) T and naïve B cells (compare Figure 3B with Figure 1B, and Supplementary Figure S3D with Supplementary Figure S1E). Human tooth appears to contain a markedly smaller population of macrophages and neutrophils than mouse tooth (‘Macro/Neut’; 46.5% versus 81.1% of the total immune cells, respectively) and a much larger population of T cells (34.7% versus 4%) (Supplementary Figure S3E). Again, one has to take into account that the included tooth samples from mouse and human differ in developmental state and turnover activity (as well as tissue type representation). For instance, the higher percentage of macrophages as observed in mouse tooth may have to do with the younger (developmental) ages included, as macrophages have been proposed to play an important role in dentinogenesis and amelogenesis processes (Park et al., 2017; Neves et al., 2020; Vieira and Modesto, 2020).

In parallel to composing the healthy tooth atlas, we established a sc transcriptome atlas of diseased human tooth, integrating the at present publicly available datasets from patients with deep caries, enamel caries, chronic apical periodontitis (CAP) and periapical granuloma (Table 4) (Lin et al., 2022; Opasawatchai et al., 2022). Following strict quality control, 17,016 cells were retained of which 3,562 and 13,454 cells were derived from dental pulp and periapical diseases (corresponding to the individual datasets), respectively (Supplementary Figures S3F,G, Table 5). Applying the established cell type markers (Table 6) revealed annotated cell clusters comparable between healthy and diseased tooth (Figure 3D, Supplementary Figures S3H,I), moreover well overlapping following integration (Figure 3E, Supplementary Figure S3J). Not unexpectedly, the diseased tooth overall seems to contain a larger proportion of immune cells than healthy tooth (52.1% versus 23.5%; Supplementary Figures S3D,K), particularly epitomized in the B cell component (compare Figures 3D,E with Figure 3B, and Supplementary Figure S3K with Supplementary Figure S3D). When stratified according to disease type, it appears that periapical pathologies (CAP and periapical granuloma) have a more pronounced immune cell component (55.8–74.9% of total cells) (thus, elicit a stronger immune cell activation and/or influx, most prominently from B cells, which may relate to their chronic nature) than carious teeth (14.4–25.1%), of which the immune cell proportion more resembles healthy human tooth (Supplementary Figure S3K). Further zooming in on those healthy tooth compartments that relate to the diseased tissue type (i.e., pulp for caries and periodontal tissue (including DF) for periodontal disease), the above-described observations hold (Supplementary Figure S3L). Furthermore, deep and enamel caries show a different composition of the immune cell component. Among others, T cell numbers appear elevated in enamel caries as compared to deep caries, whereas the latter shows a large increase in naïve B cells (Supplementary Figure S3L). This observation correlates with previous findings that the immune response elicited by caries (i.e., as recapitulated in the early enamel caries) is initially driven by T cells, and only in a later, more advanced, stage (i.e., as recapitulated in deep caries) enhanced by B cells (Izumi et al., 1995; Hahn and Liewehr, 2007; Farges et al., 2015). Thus, although the number of diseased tooth samples currently available is limited and more datasets are needed for definitive conclusions, our new inclusive human tooth atlas shows valuable potential for exploring tooth disease and differences between disease subtypes. Also, it will enable to dive deeper into human tooth biology as further demonstrated below. Overall, the composed healthy and diseased tooth atlas may serve as a roadmap for future studies that capture various (additional) human dental tissues, moreover from a variety of different pathologies, and as a tool to compare the response of the different tooth components to a wide range of noxious impacts.

### 3.5 Scrutinizing the diversity of late-stage human dental epithelium using the healthy tooth atlas

**FIGURE 4:**
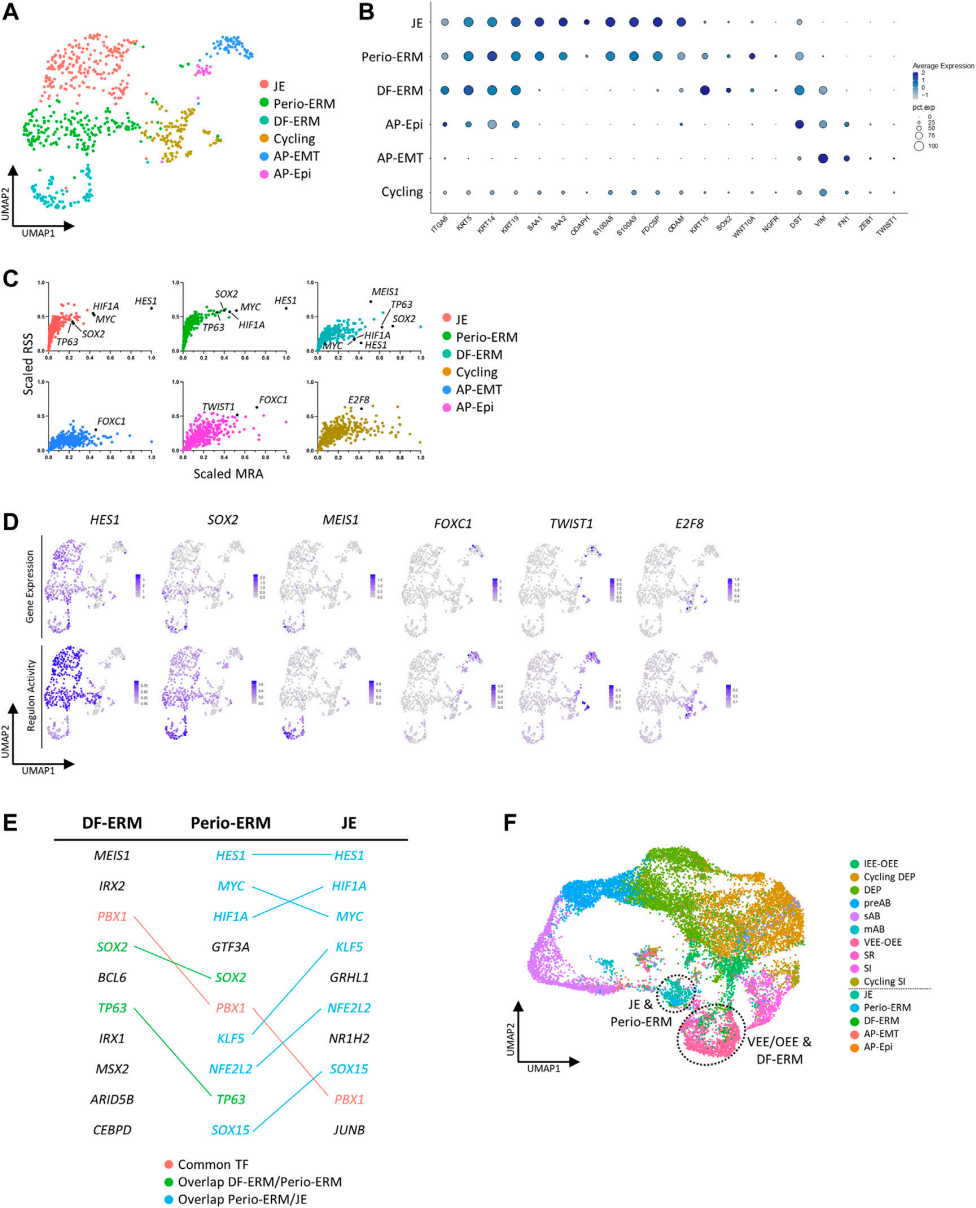
Subclustering of human tooth epithelial cells. **(A)** Annotated UMAP plot of subclustered ‘Epithelial’ and ‘Cycling’ clusters. **(B)** Dotplot displaying the percentage of cells (dot size) expressing key marker genes of the different epithelial subclusters (average expression levels indicated by color intensity). **(C)** Scatter plots (per annotated epithelial cell cluster, color-coded) of scaled RSS and MRA with each dot corresponding to an individual regulon. Black dots indicate the marked regulons. **(D)** Feature plots of gene expression (top row) and regulon activity (bottom row) of indicated TF/regulons in the epithelial subclusters. Average expression and activity levels are indicated by color intensity (see scale). **(E)** Table showing the top 10 regulon TFs for DF-ERM, perio-ERM and JE as identified through combined RSS and MRA (RSS*MRA) analysis. The overlapping regulatory landscape is mapped and color-coded to annotate common TFs (red), TFs present in both DF-ERM and perio-ERM (green) and TFs common to perio-ERM and JE (blue). **(F)** UMAP plot of integrated mouse and human DE. Dotted lines indicate co-clustering of human DF-ERM and mouse VEE-OEE, and of perio-ERM and JE.

We took advantage of the newly established healthy human tooth atlas to further resolve the DE compartment, at present not well explored and understood. Subclustering of this population, including the neighboring cycling cluster also expressing *KRT14* and *ODAM* (Figure 3B, Supplementary Figure S3D), revealed six cell groups (Figure 4A) by marker expression (Figure 4B) and DEG profile (Supplementary Figure S4A), largely separated by tissue of origin (corresponding to dataset) (Supplementary Figure S4B). The periodontal DE could be subdivided into the early-developmental epithelial cell rests of Malassez (ERM) within the DF (DF-ERM), the more mature ERM within the periodontium (Perio-ERM) and the junctional epithelium (JE) (i.e., the odontogenic epithelium-derived interface between tooth surface and gingiva (Yajima-Himuro et al., 2014)). In addition, the AP-lining epithelium could be discriminated into an epithelial part and an epithelial-to-mesenchymal transition (EMT)-associated cell group (AP-Epi and AP-EMT, respectively). The cycling cluster was comprised of cells from all source tissues, suggesting a certain degree of proliferative activity or turnover in these tooth epithelial compartments.

Intriguingly, expression overlap was observed between JE, Perio-ERM and DF-ERM, with Perio-ERM displaying intermediate characteristics of both JE and DF-ERM (Figure 4B, Supplementary Figure S4A). Along the same line, regulon analysis showed that, within the top 10 regulons for each cluster (identified based on a per cluster combination of RSS and MRA), multiple regulons are shared, with Perio-ERM again displaying an intermediate regulatory landscape. For instance, JE and Perio-ERM show high *HES1, MYC* and *HIF1A* regulon activity, while Perio-ERM and DF-ERM display high *SOX2* and *TP63* activity (Figures 4C–E). These hints of connection between JE and ERM are remarkable since both cell types have been attributed separate developmental origins as well as distinct biological functions. While ERM has been reported to be involved in enamel, cementum and PDL regeneration following tooth injury and inflammation, JE is ascribed an important role in attaching the oral gingival epithelium to the tooth surface as well as in providing protection to the constant microbial challenge from the oral cavity (Hamamoto et al., 1996; Davis, 2018; Hermans et al., 2021; Fischer and Aparicio, 2022). Whereas the ERM developmentally results from disintegration of Hertwig’s epithelial root sheath (HERS), JE is considered to develop upon tooth eruption from the reduced enamel epithelium (REE), the layer of mAB and OEE covering the developed enamel before eruption (Supplementary Figure S4C) (Luan et al., 2006; Kato et al., 2019). Our observation that perio-ERM displays an intermediate transcriptional and regulatory profile between DF-ERM and JE may indicate that the developmental origins of ERM and JE are not as distinct as previously thought, and advances the possibility that HERS-derived ERM contributes to JE formation and/or REE to ERM development (and then further to JE) (Supplementary Figure S4C), both not excluded by current knowledge. To further explore this hypothesis, we integrated all identified DE clusters across human and mouse. Interestingly, whereas perio-ERM and JE clustered together, human DF-ERM grouped with mouse VEE-OEE which gives rise to REE, further strengthening the possibility that REE may also contribute to ERM and/or vice versa (Figure 4F). Moreover, the VEE-OEE enriches for stem cells (Figure 1E) which is in line with the ERM being proposed, and recently supported, to contain DESC (Xiong et al., 2012; Tsunematsu et al., 2016; Hemeryck et al., 2022). Lineage tracing studies in mice will be essential to support these hypotheses and to definitively determine the developmental origins of JE and ERM.

Taken together, the newly composed atlas, in concert with advanced bioinformatic tools, may shed new light on the still poorly understood epithelial component of the human tooth regarding both origin and function. Moreover, this establishment of healthy and diseased tooth atlases provides a roadmap for future human tooth scRNA-seq studies, allowing to integrate and/or project newly generated data (e.g., of other tooth types or diseases, or at different stages in development) onto the consolidated maps.

### 3.6 Exploiting the tooth atlases to unravel cell-cell signaling interactions

Sc transcriptomic data analysis can be enriched using advanced computational tools that have become available in recent years (and are continuously being further developed). As an important illustration, we applied CellPhoneDB that enables to infer potential signaling interactions between a tissue’s cell types (Efremova et al., 2020).

**FIGURE 5:**
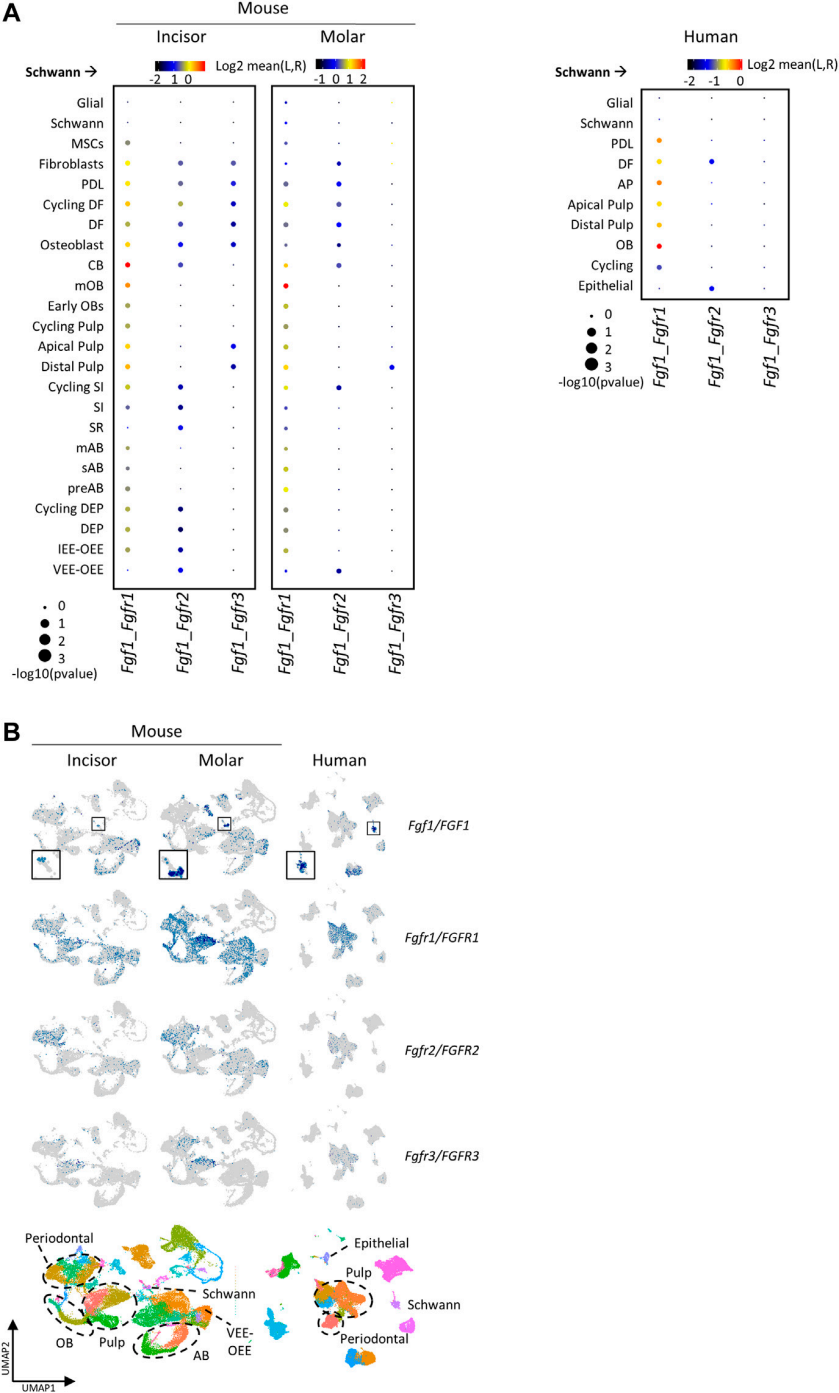
Cell-cell signaling analysis projecting FGF1 as messenger from Schwann to other cell types in human and mouse tooth. **(A,B)** Dotplot of selected ligand-receptor interactions originating from the Schwann cell cluster, identified by CellPhoneDB analysis, in mouse (left) and human (right). Size of dots indicates the *p* value of the projected interaction, and color intensity the means of the average expression levels of ligand (L) in the Schwann cell cluster and receptor (R) in the other clusters. **(B)** Gene expression plots of identified ligands/receptors in mouse and human (top), and mouse and human tooth atlases with relevant clusters highlighted (bottom).

Since Schwann cells have been described to play crucial roles during tooth development (i.e., by giving rise to dental MSC and OB) and regeneration (i.e., by co-orchestrating the pulpal inflammatory response and peripheral nerve repair) (Kaukua et al., 2014; Couve and Schmachtenberg, 2018), we focused on potential ligand-receptor interactions originating from the Schwann cell cluster towards the various DE and DM cell subsets. Interestingly, both in mouse and human, *Fgf1*-*Fgfr1* interactions between Schwann cells and the majority of annotated cell types were projected, as well as *Fgf1*-*Fgfr2* and *Fgf1*-*Fgfr3* interactions although with more distinct clusters, all with *Fgf1* originating from the Schwann cells (Figure 5A). FGF signaling has been well documented in various stages of (embryonic) tooth development and homeostasis, in particular between DE and DM (Du et al., 2018). For instance, FGF3 and FGF10 are DM signals regulating DE development and maintenance of the incisor DESC niche, while FGF4 and FGF9 from the DE are crucial regulators of DM development (Kettunen and Thesleff, 1998; Kettunen et al., 2000; Du et al., 2018). Previously, *Fgf1* has been shown to promote differentiation of OB and self-renewal of DESC (Unda et al., 2001; Chang et al., 2013). Here, our analysis prompts to hypothesize a role for FGF1 signaling also in postnatal tooth homeostasis, emanating from Schwann cells. In accordance, a recent sc analysis of peripheral nerves also identified high expression of *Fgf1* in Schwann cells compared to the neurons and mesenchymal cells in the nerves (Toma et al., 2020).

*Fgf1*-*Fgfr1* signaling activity appears lower in mouse VEE-OEE and human epithelium, both instead characterized by higher *Fgf1*-*Fgfr2* activity (Figure 5A). Curiously, both *Fgf1* and *Fgfr1* appear more expressed in molars than incisors (Figure 5B). Moreover, *Fgfr1* appears to be the predominant FGFR expressed in the tooth, detected in most cell types (Figure 5B), whereas *Fgfr2* and *Fgfr3* are mainly found in DM clusters (albeit at lower levels in human tooth, and *Fgfr2* expression also present in some epithelial cell types). Remarkably, *Fgfr2* and *Fgfr3* show non-overlapping expression patterns within the mouse DM, with *Fgfr2* predominantly found in periodontium and *Fgfr3* expressed by apical and distal pulp. Of note, mutations in all three FGF receptors are associated with dental defects in various congenital diseases, such as osteoglyphonic dysplasia (mutations in *FGFR1*), Apert syndrome (in *FGFR2*) and lacrimo-auriculo-dento-digital syndrome (in *FGFR2* and *FGFR3*) (Rohmann et al., 2006; Lu et al., 2016; Marzin et al., 2020). Moreover, *Fgfr1* and *Fgfr2* were also demonstrated to be necessary for proper odontogenesis in mouse (Takamori et al., 2008; Hosokawa et al., 2009).

Taken together, cell-cell signaling inference using the developed tooth atlases exposed FGF signaling as candidate Schwann cell-initiated regulatory niche process conserved in mouse and human tooth, thereby further illustrating the important potential of the newly composed inclusive tooth atlases when subjected to state-of-the-art (or newly developed) bioinformatic tools.

## 4 Conclusion

By re-assessing and combining recently created scRNA-seq datasets from mouse and human teeth, we established comprehensive sc atlases capturing the broad cellular diversity of the tooth spanning health and disease. Moreover, we make the composed atlases, as well as a set of subclustered compartments, available online to the dental research community in a highly accessible format by providing the loom files which can be uploaded into SCope (available from https://scope.aertslab.org) (Davie et al., 2018), an online application allowing to map the separate clusters and interrogate expression of genes of interest. Taken together, the atlases will provide a powerful resource and tool for the dental research community. In addition to being a strong activator or accelerator of hypothesis-driven research, i.e. exposing or supporting novel hypotheses which can then further be experimentally investigated, the atlases can be used as a technical resource and roadmap for future tooth scRNA-seq studies, allowing integration of newly generated data which can lead to enriched insights. Also, further developments in bioinformatic programs, as well as in sc (multi-)omics technologies enabling the (co-)assessment of the sc epigenome, metabolome and proteome and the inclusion of spatial information (spatial transcriptomics), will undoubtedly highly advance our understanding of fundamental tooth biology, with significant translational and clinical implications. In particular, further deep unraveling of the tooth cellular and molecular landscape will pave the way to tissue engineering and the (re-)construction of a biological or biomimetic tooth, which essentially relies on detailed knowledge of needed cell types and extracellular matrix players.

## Data Availability

Publicly available datasets were analyzed in this study. This data can be found here: GSE131204 (GEO, https://www.ncbi.nlm.nih.gov/geo/query/acc.cgi?acc=GSE131204), GSE120108 (GEO, https://www.ncbi.nlm.nih.gov/geo/query/acc.cgi?acc=GSE120108), FB00001104 (FaceBase, https://www.facebase.org/chaise/record/#1/isa:dataset/RID=1-RK82), GSE146855 (GEO, https://www.ncbi.nlm.nih.gov/geo/query/acc.cgi?acc=GSE146855), GSE146123 (GEO, https://www.ncbi.nlm.nih.gov/geo/query/acc.cgi?acc=GSE146855), FB00001105 (FaceBase, https://www.facebase.org/chaise/record/#1/isa:dataset/RID=1-S7GJ), GSE167989 (GEO, https://www.ncbi.nlm.nih.gov/geo/query/acc.cgi?acc=GSE167989), GSE168450 (GEO, https://www.ncbi.nlm.nih.gov/geo/query/acc.cgi?acc=GSE168450), GSE160358 (GEO, https://www.ncbi.nlm.nih.gov/geo/query/acc.cgi?acc=GSE160358), GSE146123 (GEO, https://www.ncbi.nlm.nih.gov/geo/query/acc.cgi?acc= GSE146123), GSE161267 (GEO, https://www.ncbi.nlm.nih.gov/geo/query/acc.cgi?acc= GSE161267), https://data.mendeley.com/datasets/7ryrp25y6z, GSE167251 (GEO, https://www.ncbi.nlm.nih.gov/geo/query/acc.cgi?acc=GSE167251), E-MTAB-10596 (ArrayExpress, https://www.ebi.ac.uk/arrayexpress/experiments/E-MTAB-10596/), GSE185222 (GEO, https://www.ncbi.nlm.nih.gov/geo/query/acc.cgi?acc= GSE185222), GSE181688 (GEO, https://www.ncbi.nlm.nih.gov/geo/query/acc.cgi?acc=GSE181688). All analysis codes will be made available in a GitHub repository after publication, accessible at https://github.com/fhermans27/scRNAseq-tooth-atlas. Loom files for interactive exploration will be made available via Mendeley Data (DOI: 10.17632/2kskdknngb.1).
