# Supplementary material for: Establishment of inclusive single-cell transcriptome atlases from mouse and human tooth as powerful resource for dental research
Source: Front Cell Dev Biol. 2022 Oct 10;10:1021459. doi: 10.3389/fcell.2022.1021459 (PMC9590651; doi:10.3389/fcell.2022.1021459)

Supplementary Material

**Supplementary Figure 1. Establishment of an inclusive mouse tooth single-cell atlas.** (A) Violin plots showing the distribution of counts, features and percent mitochondrial genes of the included datasets before and after performing quality control (QC) on each dataset individually. Cut-off values used are indicated in Supplementary Table 2. (B) Pie charts showing the total number of cells derived from each dataset or tooth type after QC. (C) Dotplot displaying the percentage of cells (dot size) expressing key marker genes with average expression levels (color intensity). (D) Examples of gene module analysis with color intensity indicating module score. (E) Pie charts showing the total number of cells in each of the 35 annotated cell types. (F) Schematic representation of the mouse incisor labial cervical loop niche, with annotated DE clusters. (G) Plots of gene expression (top rows) and individual Nebulosa expression densities (bottom rows) of indicated DESC genes.

**Supplementary Figure 2. Subclustering of the mouse ameloblast lineage.** (A) Dotplot displaying the percentage of cells (dot size) expressing predicted regulon target genes with average expression levels (color intensity). (B) Top 20 GO terms for each cluster of pseudotime-ordered genes ranked and colored by *P* value.

**Supplementary Figure 3. Establishment of an inclusive human tooth single-cell atlas.** (A) Violin plots showing the distribution of counts, features and percent mitochondrial genes of the included datasets before and after performing quality control (QC) on each dataset individually. Cut-off values used are indicated in Supplementary Table 5. (B) Pie charts showing the total number of cells derived from each dataset and tissue type after QC. (C) Dotplot displaying the percentage of cells (dot size) expressing key marker genes with average expression levels (color intensity). (D) Pie charts showing the total number of cells in each of the 23 annotated cell types. (E) Bar graphs indicating the percentage of immune cells in relation to total cell number (left), and the percentage of different immune cell types in relation to the total number of immune cells (right) for mouse and human tooth. (F) Violin plots showing the distribution of counts, features and percent mitochondrial genes of the included datasets before and after performing QC on each dataset individually. Cut-off values used are indicated in Supplementary Table 8. (G) Pie charts showing the total number of cells derived from each dataset and pathology after QC. (H) UMAP representation of disease subtypes (left) and dataset/tissue type (right). (I) Dotplot displaying the percentage of cells (dot size) expressing key marker genes with average expression levels (color intensity). (J) UMAP plots showing tissue type (left), dataset (middle) and health/disease condition (right) for the integrated atlas of healthy and diseased tooth. (K) Pie charts showing the total number of cells in each of the 23 annotated cell types of diseased tissue, and bar graph depicting the percentage of immune cells in relation to the total cell number for healthy and disease subtype. (L) Bar graphs indicating the percentage of each immune cell type in relation to the total number of immune cells (right) for healthy and disease subtype.

**Supplementary Figure 4. Subclustering of human tooth epithelial cells.** (A) Dotplot displaying the percentage of cells (dot size) expressing selected differentially expressed genes (DEG) with average expression levels (color intensity) (left), and gene expression plots of selected DEG (right). (B) UMAP plot of subclustered ‘Epithelial’ and ‘Cycling’ clusters mapping the ‘dataset - tissue type’. (C) Currently proposed (top) and hypothesized (bottom) developmental origins of ERM and JE.


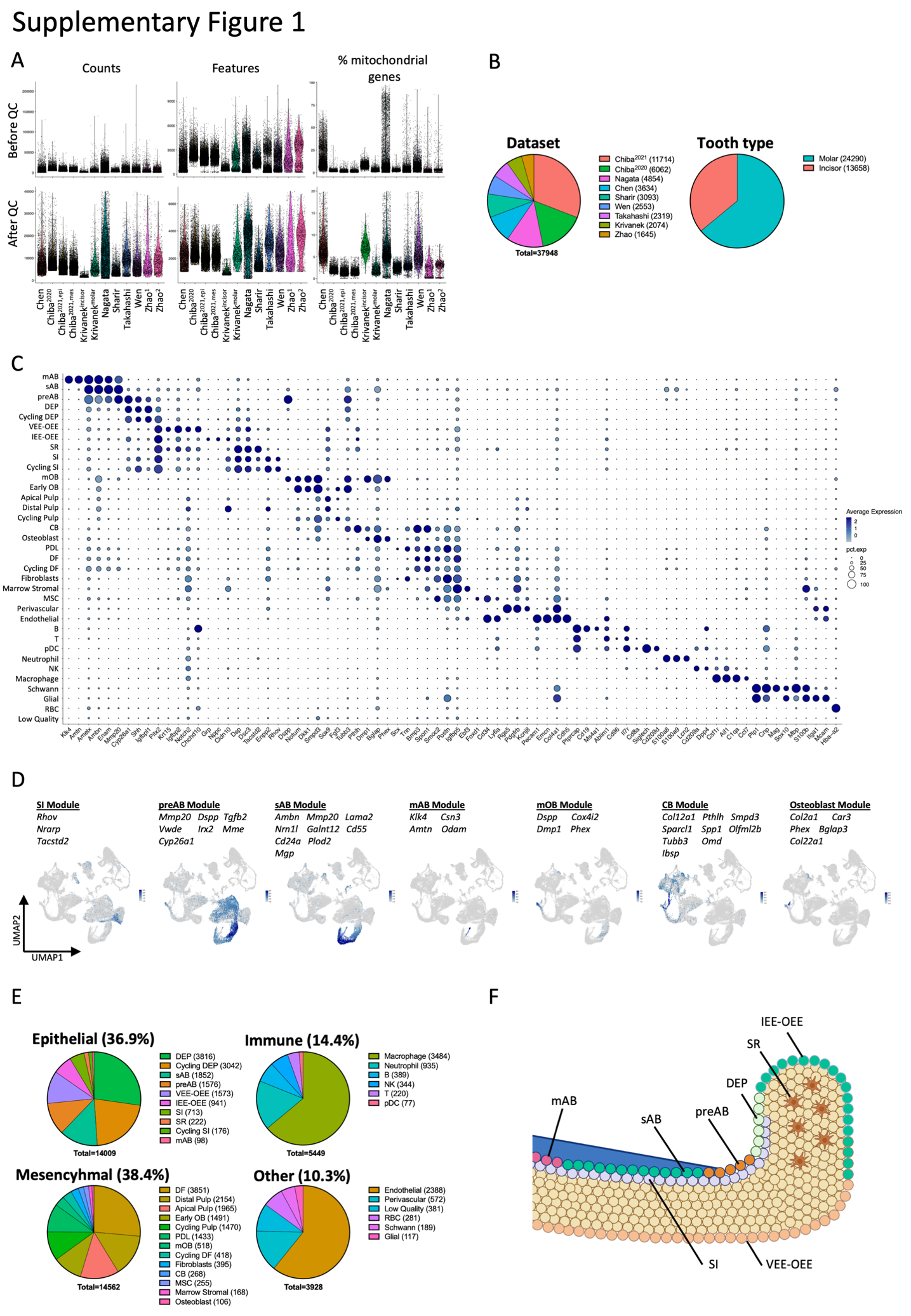


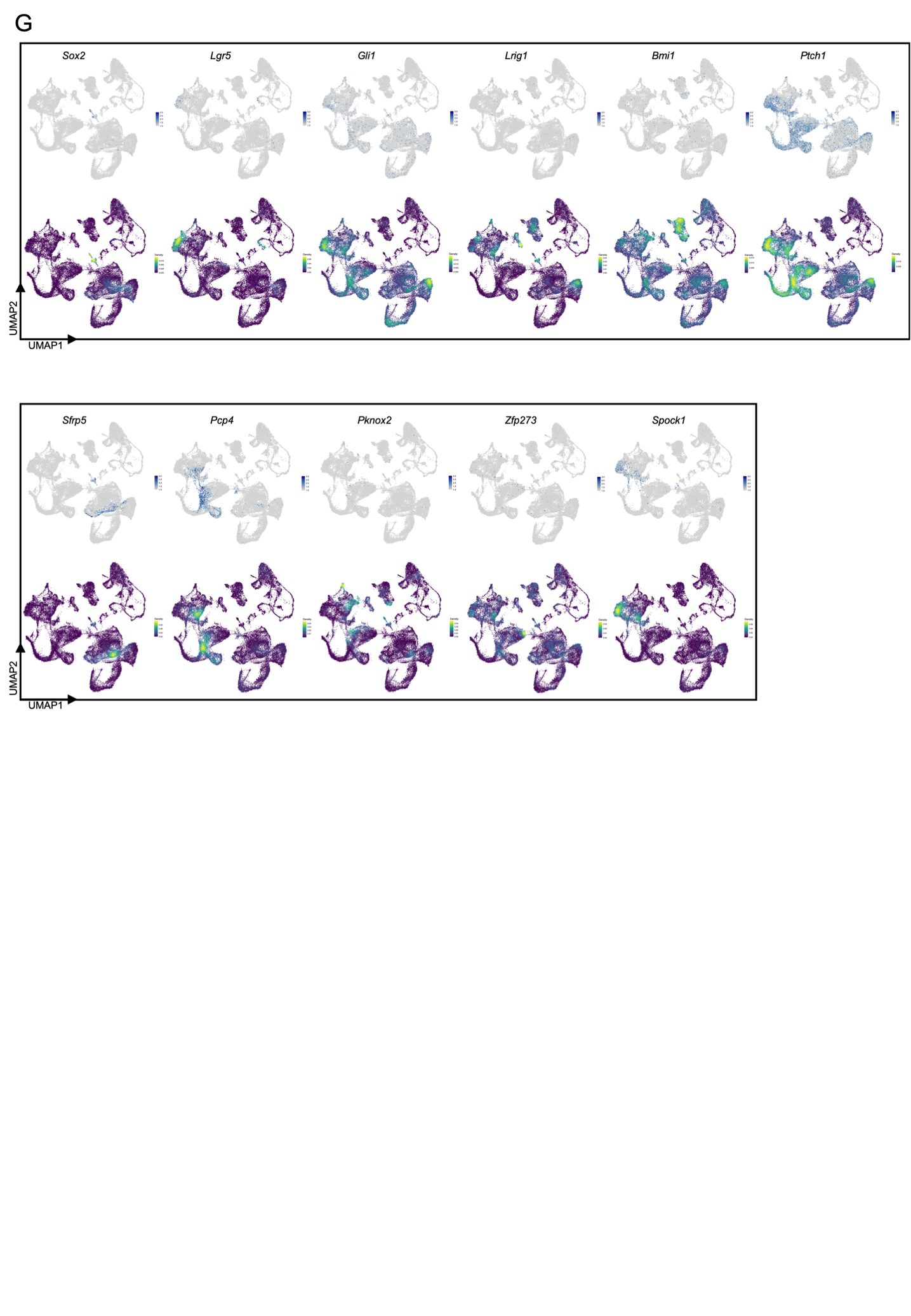


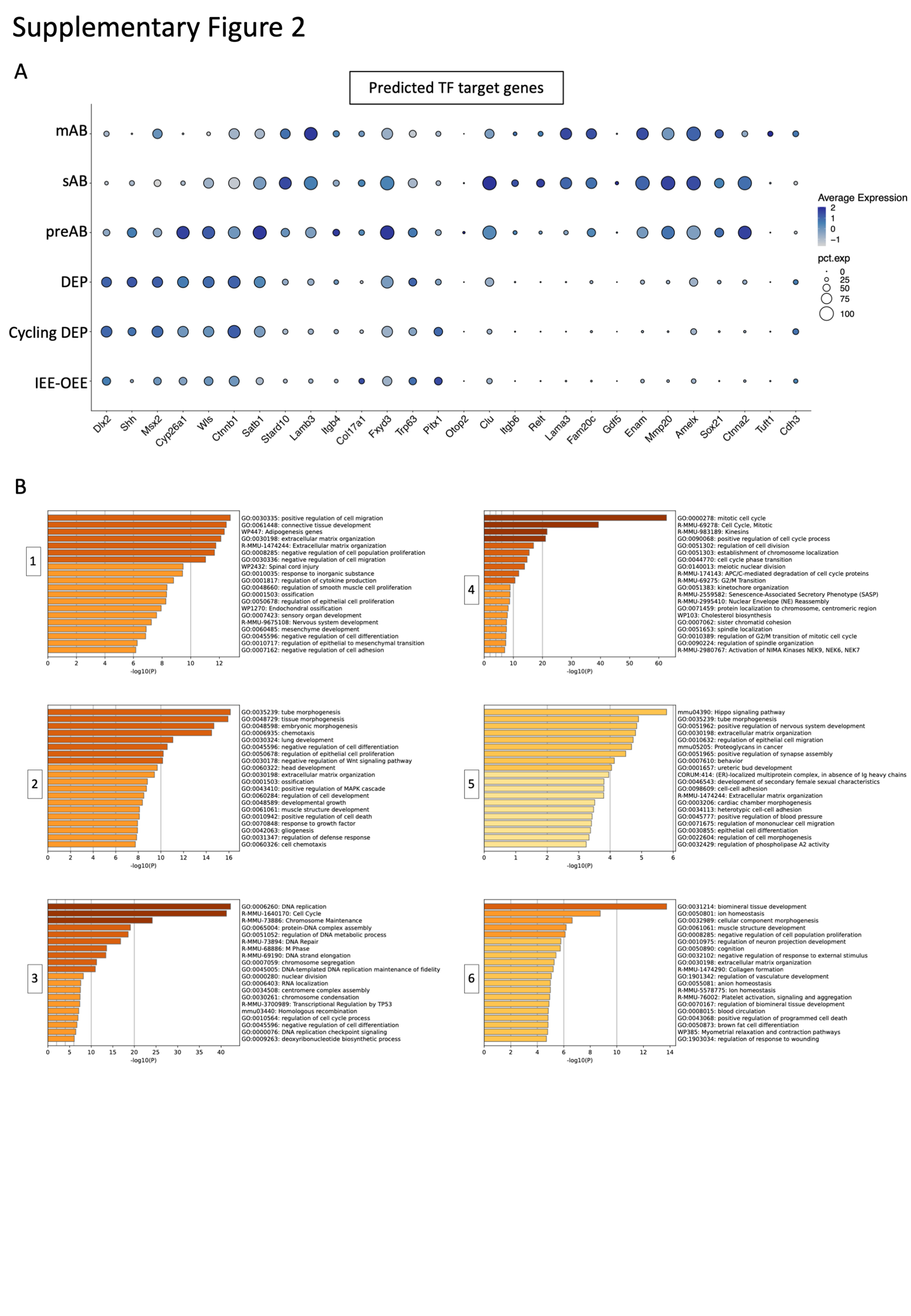


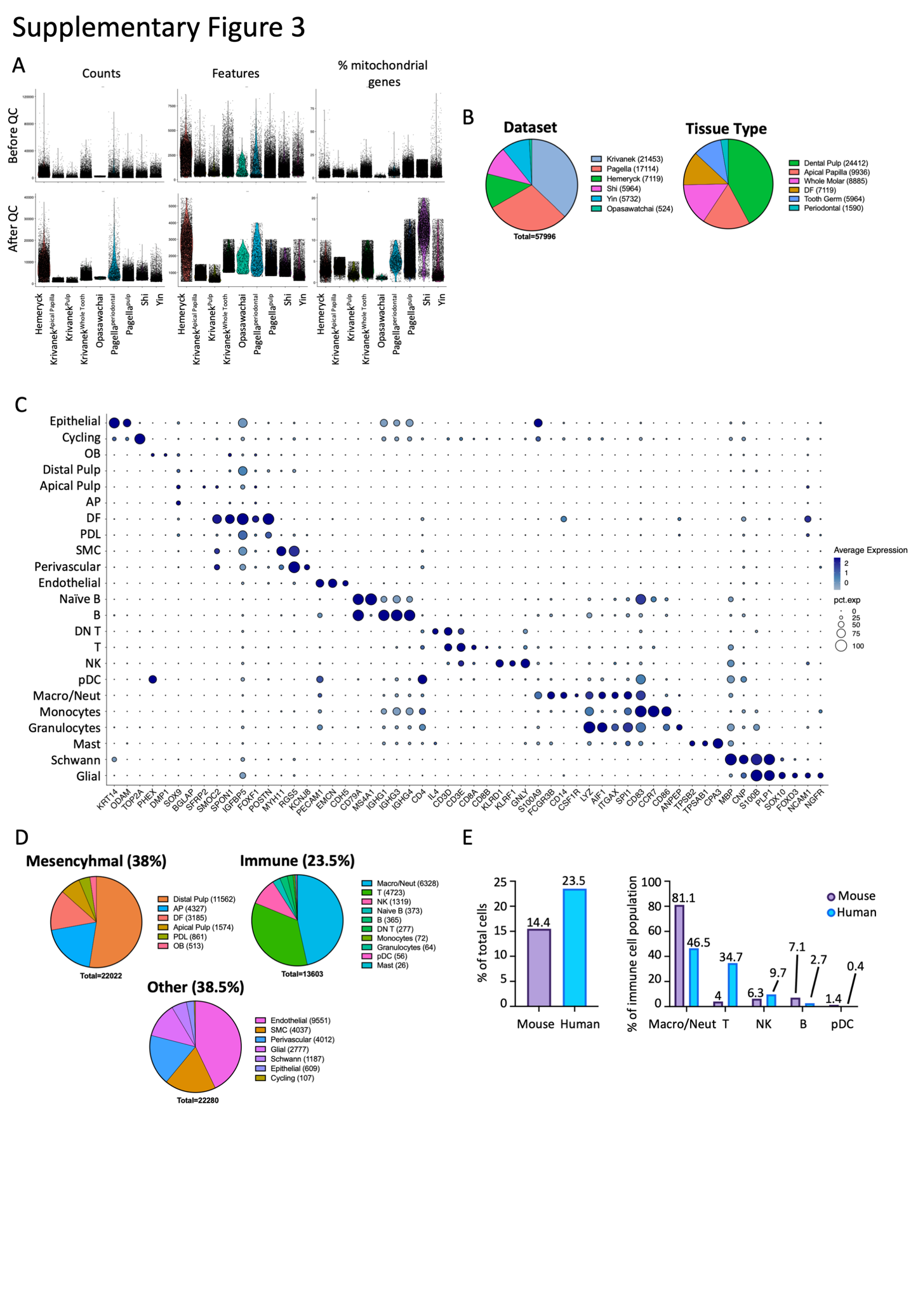


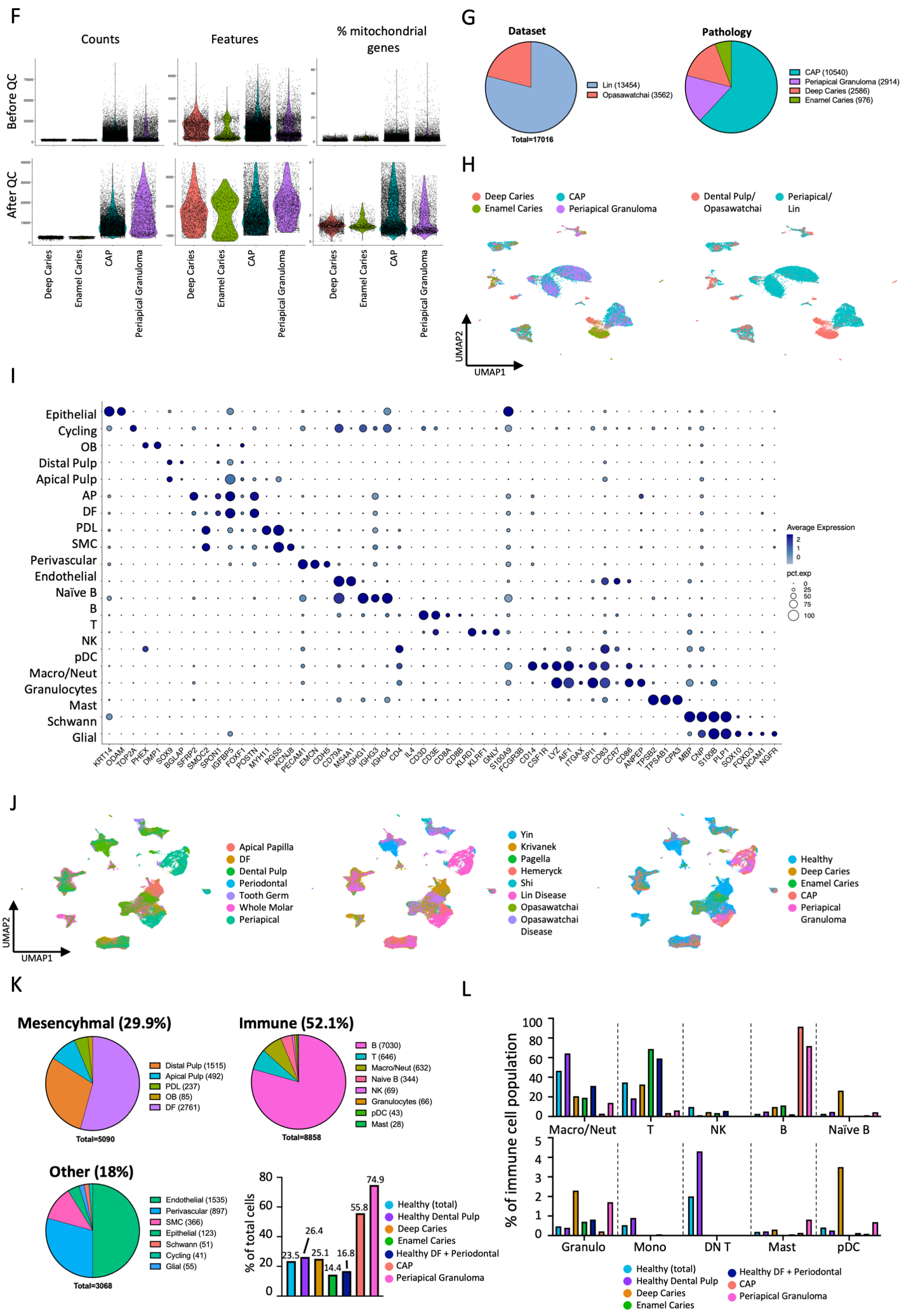


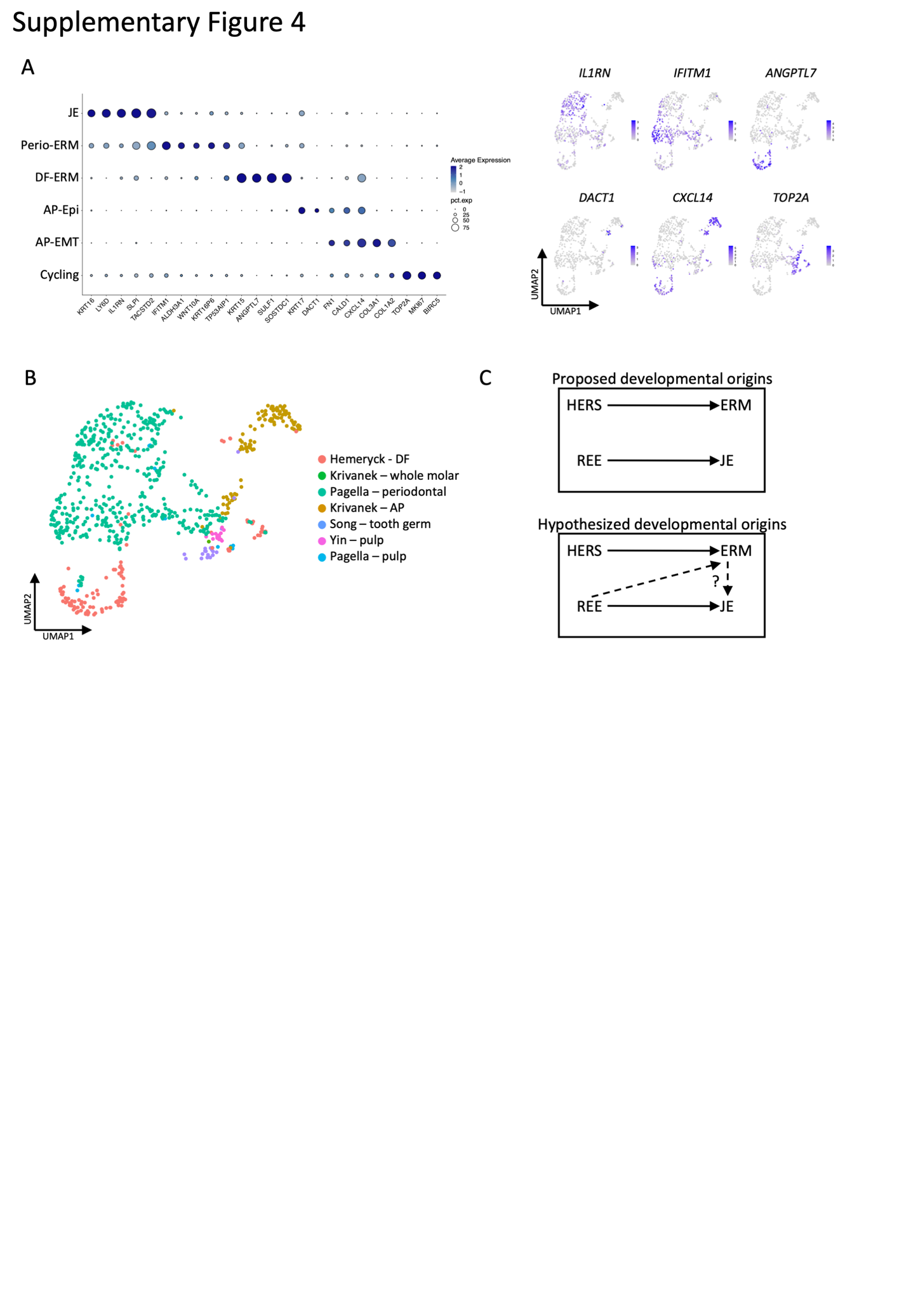

Supplement: Supplementary file 1 [file DataSheet1.ZIP › Hermans - Tooth Atlas - FrontCellDevBiol - supplementary materials.docx]
